# Whole blood transcriptome profiling identifies candidate genes associated with alopecia in male giant pandas (*Ailuropoda melanoleuca*)

**DOI:** 10.1186/s12864-022-08501-z

**Published:** 2022-04-12

**Authors:** Haibo Shen, Caiwu Li, Ming He, Yan Huang, Jing Wang, Jing Luo, Minglei Wang, Bisong Yue, Xiuyue Zhang

**Affiliations:** 1 Key Laboratory of Bio-Resources and Eco-Environment, Ministry of Education, College of Life Science, Sichuan University, Chengdu, 610064 PR China; 2 Key Laboratory of State Forestry and Grassland Administration On Conservation Biology of Rare Animals in The Giant Panda National Park, China Conservation and Research Center for the Giant Panda, Dujiangyan, 611830 Sichuan PR China; 3 Sichuan Key Laboratory of Conservation Biology On Endangered Wildlife, College of Life Sciences, Sichuan University, Chengdu, 610064 PR China; 4 No. 24 South Section 1, Yihuan Road, Chengdu, 610065 Sichuan China

**Keywords:** Giant panda, Alopecia, Peripheral blood, Transcriptome profiling, Differential gene expression

## Abstract

**Background:**

The giant panda (*Ailuropoda melanoleuca*) is a threatened species endemic to China. Alopecia, characterized by thinning and broken hair, mostly occurs in breeding males. Alopecia significantly affects the health and public image of the giant panda and the cause of alopecia is unclear.

**Results:**

Here, we researched gene expression profiles of four alopecia giant pandas and seven healthy giant pandas. All pandas were approximately ten years old and their blood samples collected during the breeding season. A total of 458 up-regulated DEGs and 211 down-regulated DEGs were identified. KEGG pathway enrichment identified that upregulated genes were enriched in the Notch signaling pathway and downregulated genes were enriched in ribosome, oxidative phosphorylation, and thermogenesis pathways. We obtained 28 hair growth-related DEGs, and identified three hub genes *NOTCH1*, *SMAD3*, and *TGFB1* in PPI analysis. Five hair growth-related signaling pathways were identified with abnormal expression, these were Notch, Wnt, TGF-β, Mapk, and PI3K-Akt. The overexpression of *NOTCH1* delays inner root sheath differentiation and results in hair shaft abnormalities. The delayed hair regression was associated with a significant decrease in the expression levels of *TGFB1.*

**Conclusions:**

Our data confirmed the abnormal expression of several hair-related genes and pathways and identified alopecia candidate genes in the giant panda. Results of this study provide theoretical basis for the establishment of prevention and treatment strategies for giant pandas with alopecia.

**Supplementary Information:**

The online version contains supplementary material available at 10.1186/s12864-022-08501-z.

## Background

The main structures of hair include the dermal papilla, bulge, sebaceous gland, and hair shaft [1]. The hair shaft is produced by a hair follicle (HF) and possesses a unique ability to regenerate itself. Morphogenesis of the HF occurs in the embryo, and postnatal follicles undergo a cycle of renewal in three phases: anagen (growth), catagen (degradation) and telogen (resting) [2]. Follicle stem cells are activated at the telogen-to-anagen transition, to initiate a new round of hair growth [1]. Many genes and signaling pathways are involved in HF development, such as Notch-1 receptor and its three ligands, Delta-1, Jagged-1 and Jagged-2 in Notch [3], Wnt10b in Wnt [4], TGF-β2 in TGF-β [5], Gab1 in Mapk [6], and FGF16, CSF3, interleukin (IL)6, and oncostatin M in PI3K-Akt [7] signaling pathways. The investigation of genes and molecular signaling in the regulation of hair follicles is ongoing.

Mammalian hair has important biological functions, including sunlight reflection or absorption, heat retention, water resistance, mate attraction, skin protection and social communication [1]. Despite these important biological functions, premature or atypical hair loss (i.e. alopecia) is common. For example, recent studies have reported that rhesus macaques [8], mice [9], Andean bears [10] and polar bears [11] displayed some extent of alopecia. Alopecia in captive female rhesus macaques occurred during gestation and parturition [8] and was associated with pregnancy and chronic stress [12]. Whereas otherwise healthy captive Andean bears acquired the slowly progressive alopecia syndrome of unknown aetiology [10]. While 28% of polar bears in the southern Beaufort Sea region had varying degrees of alopecia that was concomitant with body condition declines [11]. Numerous factors may contribute to alopecia, ranging from naturally occurring processes (seasonality, aging) to various diseases (stress, endocrine disorders, genetic mutations, immunologic diseases, bacterial and fungal infections) [13, 14]. Alopecia is not fully understood but it is a common and complex phenomenon in captive mammals [15].

The endemic giant panda (*Ailuropoda melanoleuca*), is a national treasure in China and a flagship species within China and internationally. The unique bamboo-specialist carnivore has become a global conservation symbol due to efforts to save the species, by overcoming difficulties in captive breeding, have been captured on film and now on social media. Humans prefer animals with physiological and behavioral similarities and these bears often display behaviors similar to human children, which are frequently comedic and therefore are loved by people across the world [16]. Akin to other captive species, the giant panda can develop alopecia and mostly occurs in breeding males in spring and summer. The alopecia, characterized by symmetrical thinning and broken hair or hair loss, is mainly concentrated on the limbs and abdomen. Occasionally pandas can become bald in certain parts of the body. Alopecia can seriously affect the health of giant pandas, but can also damage the public image of the flagship species.

At present, the causes of giant panda alopecia are unclear and might be complex. It is likely that mapping and analyzing genes involved in alopecia will assist with understanding the cause and may provide avenues for treatment. Blood is the main component of the animal immune system and plays a key role in the animal immune system. At the same time, the gene expression of blood cells that circulate through various tissues and organs can experience unique changes due to disease or injury in other tissues and cells of the body. Additionally, many genes previously thought to be expressed only in non-blood tissues are also expressed in peripheral blood cells [17]. Therefore, the phenotype of blood cell can reflect the physiological and pathological events of other tissues, and different blood markers have been widely used as substitutes for organs to monitor their health status [18]. Next-generation sequencing analysis has greatly improved genetic diagnostics and therefore, we aimed to mine the differentially expressed genes (DEGs) in male giant panda peripheral blood with and without alopecia and discuss the possible cause of giant panda alopecia.

## Material and methods

### Samples

We collected peripheral blood samples from 11 captive adult male giant pandas (9 to 11 years old) from April to June. These 11 pandas were housed in the China Conservation and Research Center for Giant Panda in Chengdu, China. Samples were taken during routine physical examinations to minimize stress and the need for additional captures (i.e. anesthesia). We divided the 11 pandas into two groups, with and without alopecia. The (with) alopecia group had four giant pandas and the normal (control) group consisted of seven pandas (Additional file 1: Table S1).

All giant pandas continued their normal captive existence after completion of the sampling and were in no way physically harmed due to our research. The China Conservation and Research Center for Giant Panda agreed to provide blood samples and they assisted with the research.

### Library preparation and sequencing

Approximately 3 ml blood was collected from each individual and immediately transferred into a paxgene blood RNA tube (BD company, USA) to stabilize the RNA in the cell and avoid degradation. Total RNA was extracted using TRIzol reagent (Invitrogen, Carlsbad, CA, USA) following the manufacturer’s protocol and treated with RNase-free DNase I. Nanodrop 8000 Spectrophotometer (Thermo scientific) was used to evaluate the purity and concentration of RNA. We confirmed RNA integrity using an RNA PicoChip with Agilent 2100 Bioanalyzer (Agilent Technologies). We then used the extracted RNA samples for the cDNA synthesis and amplified double-stranded cDNA. We used kits from the Illumina Company to perform sequencing libraries construction, quality control and quantification as per manufacture's recommendations. We sequenced the cDNA library on the Illumina sequencing platform (HiSeq 2000) according to standard procedures. The Genome Analyzer Pipeline version 2.0 ran the image analysis and base-calling using default parameters. Our sequencing generated 150-bp paired-end reads.

### Quantification and mapping

All raw reads were processed with adapter trimming and reads filtering by trim-galore version 0.5.0 (http://www.bioinformatics.babraham.ac.uk/projects/trim_galore/) to ensure data quality control. We used FastQC version 0.11.5 (http://www.bioinformatics.babraham.ac.uk/projects/fastqc/) to generate quality reports thus ensuring that clean data were used in subsequent analyses. We built a genome index and mapped reads data on the giant panda reference genome using HISAT2 version 2.1.0 [19]. The giant panda reference genome (v90) and reference annotation were downloaded from the Ensembl website (ftp://ftp.ensembl.org/pub/release-90/).

### Calculating differentially expressed genes (DEGs)

The alignment program HISAT2 generated SAM files that stored mapping information and these files were sorted by SAMtools version 1.7 [20]. We obtained a sample read counts file from BAM files using featureCounts version v1.6.2 [21], allowing quicker counting of features. Reads counts were used to calculate the expression value of transcripts per million (TPM). The data matrix of the number of reads was analyzed using principal components analysis (PCA) in R function prcomp. A PCA plot with clustering information was illustrated by R package ggbiplot (https://github.com/vqv/ggbiplot). The counts file was entered into R package edgeR [22] that identified genes with significant expression differences based on the Poisson model. We calculated the expression fold change (FC) and false discovery rate (FDR) to identify differentially expressed genes (DEGs) in the two groups. We set a cut-off of 1 for |log_2_FC| and 0.05 for FDR. The filtered genes were retained for further analysis.

### Analysis of gene enrichment

Genes were annotated and analyzed using the Gene Ontology (GO) and Kyoto Encyclopedia of Genes and Genomes (KEGG) databases to obtain known functions of these gene sets. We compared our dataset with three GO categories; molecular function, biological process and cellular component. Genes were clustered to GO terms using a web server g:Profiler [23]. We calculated the FDR using g:SCS algorithm and a threshold of 0.05 was set for FDR. The KEGG pathway map analysis was performed by KOBAS 2.0 [24] and this analysis presented biological interpretation of higher-level systemic functions,.

### Analysis of protein–protein interaction network

We input DEGs into the STRING [25] database of known and predicted protein–protein interactions to obtain protein–protein interaction networks. Protein–protein interaction outputs were simple texts in tabular form. We used Cytoscape [26] to visualize molecular interactions and a Cytoscape plugin, cytoHubba [27], to calculate hub genes. The EcCentricity algorithm was used to calculate hub genes. Betweeness, closeness, and degree topological algorithms were used for verification.

### Real-time quantitative PCR (qRT-PCR)

Real-time quantitative PCR (qRT-PCR) was performed to confirm the expression changes of genes and six genes were selected randomly. We used the same reference gene, GAPDH [28], as previous studies. The real-time quantitative PCR was performed with six samples (A1, A2, A3, N1, N2, N3) because RNA of several samples had already been used. The sequences of primers were predicted using Primer-BLAST [29] and were synthesized at TSINGKE (listed in Additional file 2: Table S2). Each qPCR reaction system was 10 μL containing 5 μL of 2 × M5 Hiper SYBR Premix EsTaq (mei5, Beijing, China), 0.2 μL cDNA, 0.2 μL of forward and reverse primer and nuclease-free water. Reactions were performed in triplicate on Bio-Rad CFX96 Touch. We used the following program: 95 °C for 30 s, followed by 40 cycles of 95 °C/10 s, 60 °C/15 s; then 72 °C for 10 s. Gene expression levels were calculated using optimized comparative Ct (2^−ΔΔCt^) value method [30]. GraphPad Prism 8.0.2 (https://www.graphpad.com/scientific-software/prism/) was used to analyze data.

## Results

### Reads sequencing and processing

**Fig. 1:**
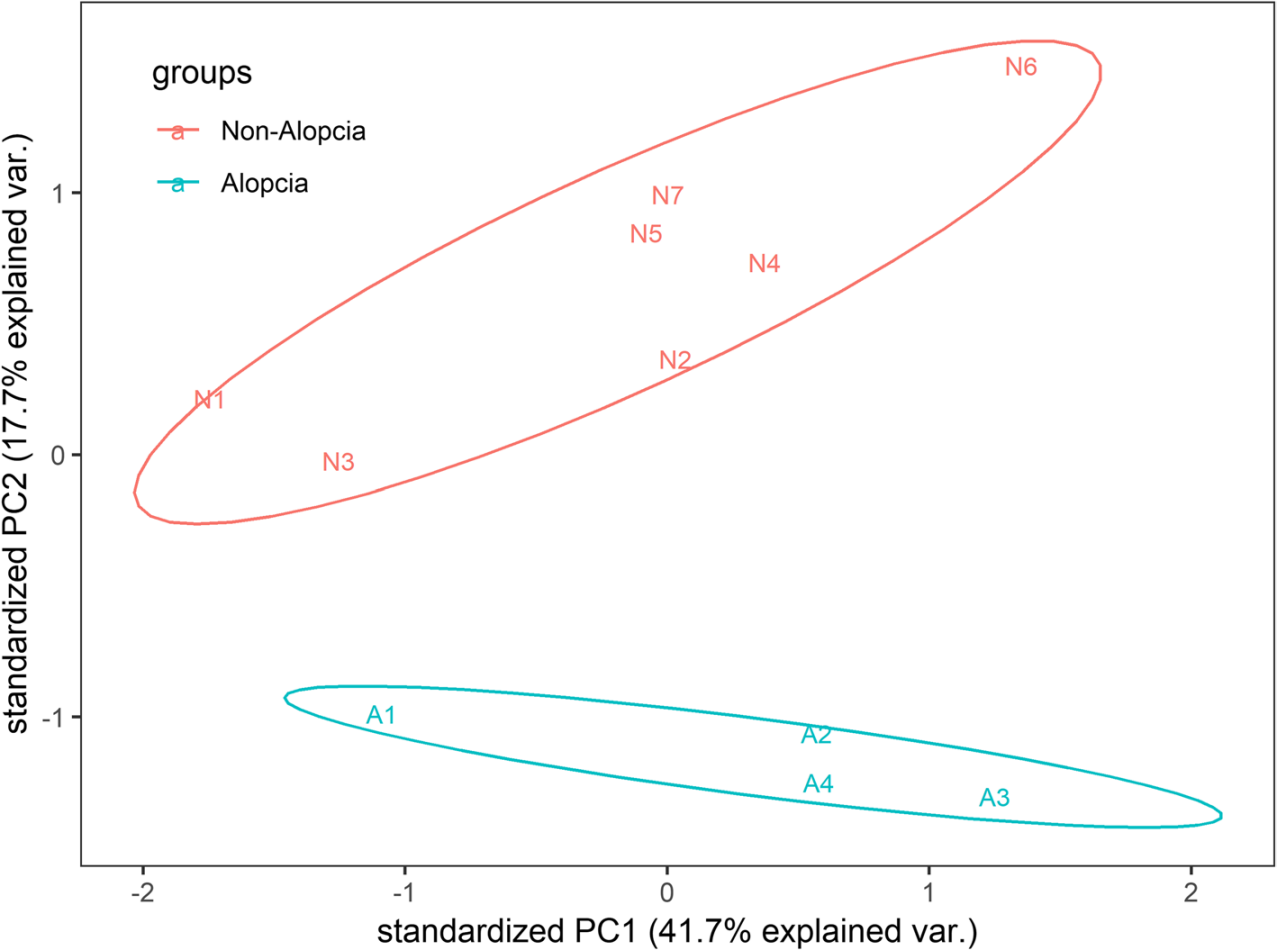
PCA of blood tanscriptome from normal and alopecia giant panda

Clean reads data were generated from raw Illumina RNA-Seq data. A total of 79.27 Gb of paired-end clean data were generated (Additional file 1: Table S1) and the percent of Q30 was greater than 85% according to FastQC. HISAT2 mapping results indicated that sample overall alignment rates were between 86 and 90%. We conducted read summarization using program featureCounts and counts were transformed into a numerical matrix. We found that the 11 samples were divided into two groups according to a PCA normalized matrix, which agreed with the groups being divided by phenotype (Fig. 1).

### Identification of DEGs

**Fig. 2:**
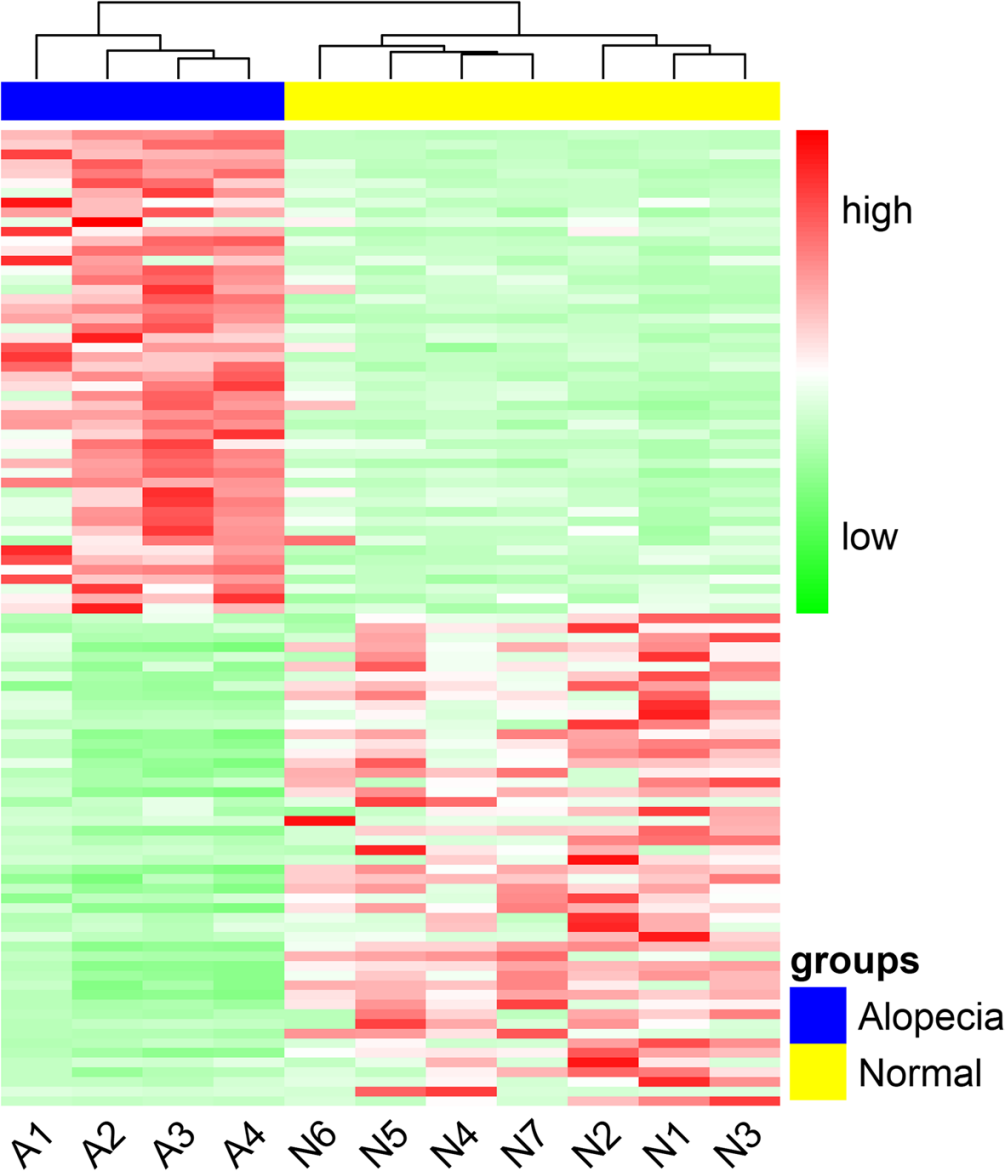
Heat map plot of top 50 up-regulated and down-regulated genes. Red color indicates genes which were up-regulated and green color indicates genes which were down-regulated

We identified 458 up-regulated DEGs and 211 down-regulated DEGs in the alopecia group compared to the normal group (Additional file 3: Table S3). Of the 458 up-regulated genes, 365 were annotated, while 140 of the 211 down-regulated DEGs had annotations. Our study was mainly based on annotated genes. We identified the most significant DEGs by sorting by log_2_FC value. We selected the top 50 up-regulated and down-regulated genes to draw the gene expression heatmap (Fig. 2).

In the top 10 up-regulated DEGs, *SHISA7* (shisa family member 7) and *ASCL5* (achaete-scute family bHLH transcription factor 5) were 10 and sixfold higher in alopecia males than normal males, respectively. Also identified in the top 10, *TMEM170B* (transmembrane protein 170B)*, IFITM5* (interferon induced transmembrane protein 5), and *FZD8* (frizzled class receptor 8) are associated with Wnt signaling. Wnt signaling participates in numerous fundamental processes, such as embryonic development and hair growth.

In the top 10 down-regulated DEGs, *KLK5* (kallikrein related peptidase 5) and *TCEA3* (transcription elongation factor A3) were 10 and sixfold lower in alopecia males than normal males, respectively. *KLK5* is highly expressed in human hair follicles and sweat glands, and is involved in keratinocyte desquamation and pigmentation. Other top 10 down-regulated DEGs included *CD52* (CD52 molecule) and *TRDC* (T cell receptor delta constant) and these are related to the immune system. *TRDC* is involved in the formation of T cell receptors. *RPL21* (ribosomal protein L21) was also a top 10 and is known to encode a ribosomal protein.

### Gene Ontology enrichment of DEGs

**Fig. 3:**
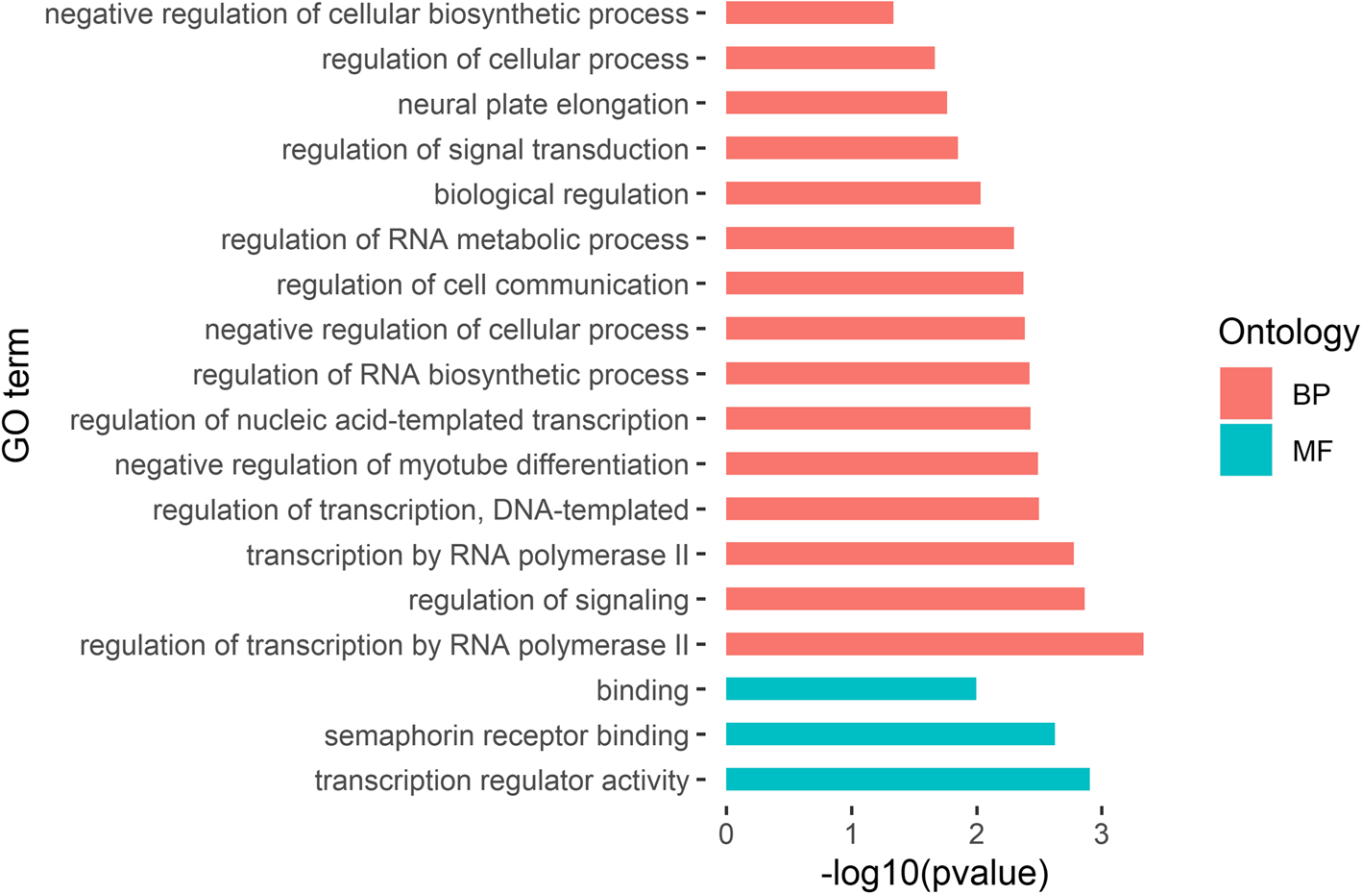
Partial GO enrichment of up-regulated DEGs

We found that up-regulated DEGs were enriched in 37 GO terms, consisting of three terms in molecular function and 34 terms in biological process (Fig. 3). The three molecular function terms were transcription regulator activity (GO:0,140,110), semaphorin receptor binding (GO:0,030,215), and binding (GO:0,005,488). The 34 biological process terms included negative regulation of myotube differentiation (GO:0,010,832), regulation of cell communication (GO:0,010,646), and regulation of response to stimulus (GO:0,048,583).

**Fig. 4:**
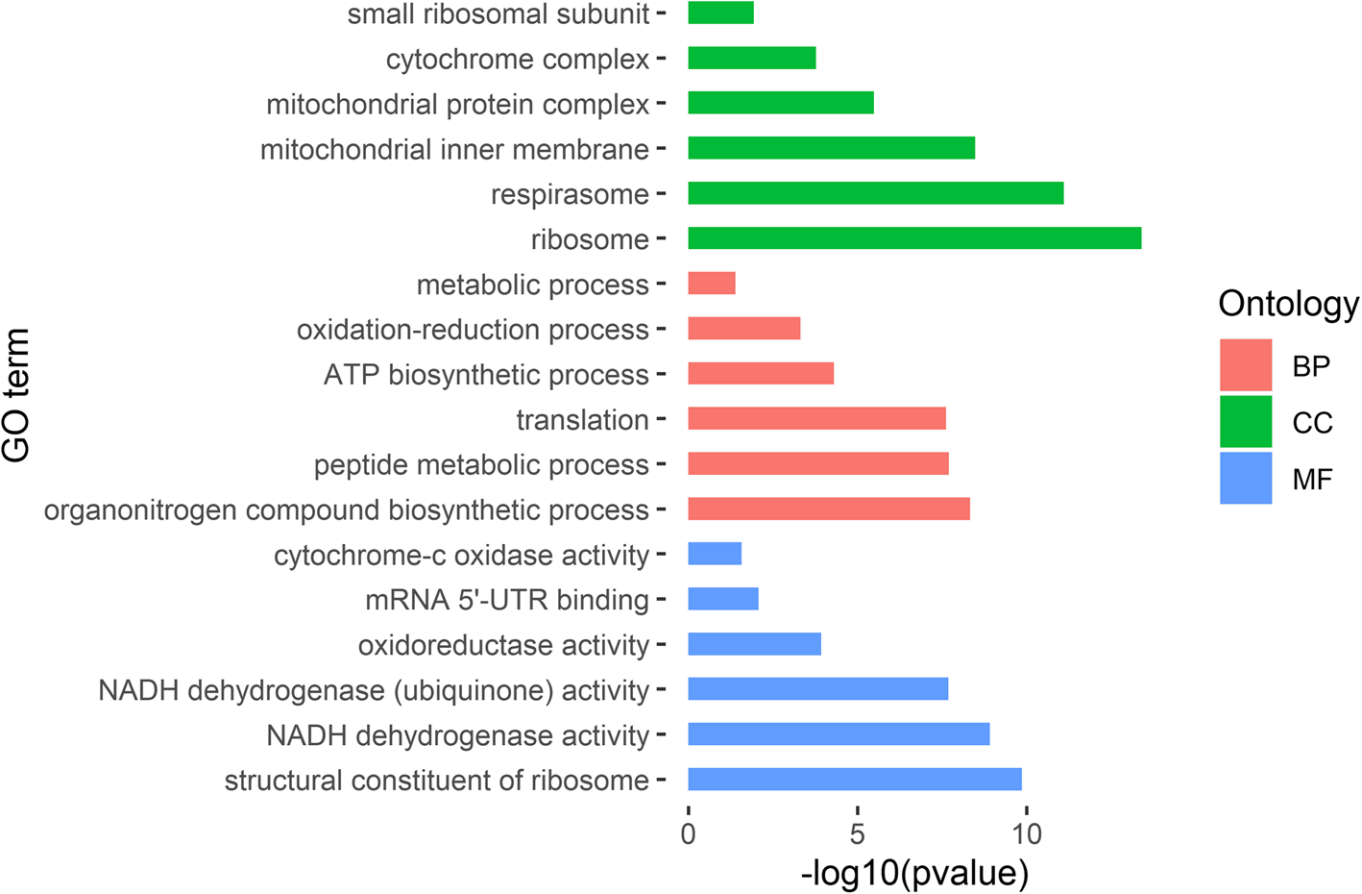
Partial GO enrichment of down-regulated DEGs

The down-regulated DEGs were enriched in 78 GO terms, with 14 terms in molecular function, 28 terms in biological process, and 36 terms in cellular component (Fig. 4). The top three biological process terms were organonitrogen compound biosynthetic process (GO:1,901,566), peptide metabolic process (GO:0,006,518), and translation (GO:0,006,412). The top three cellular component terms were ribosome (GO:0,005,840), respirasome (GO:0,070,469), and mitochondrial inner membrane (GO:0,005,743). The terms in molecular function mainly included structural constituent of ribosome (GO:0,003,735), NADH dehydrogenase activity (GO:0,003,954), mRNA 5'-UTR binding (GO:0,048,027), and cytochrome-c oxidase activity (GO:0,004,129). All GO term enrichments are shown in Additional file 4: Table S4.

### Pathway enrichment of DEGs

**Fig. 5:**
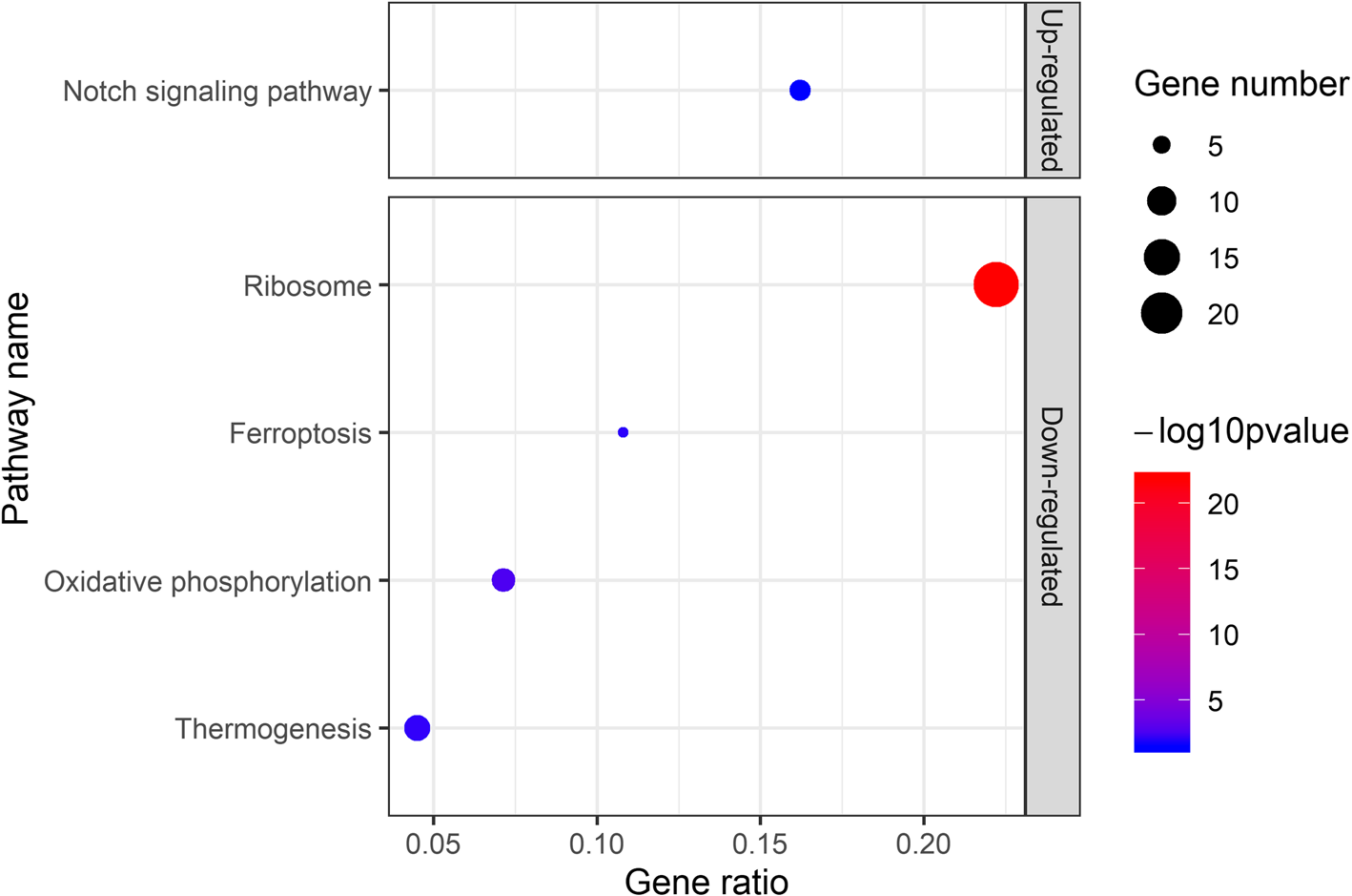
KEGG enrichment of up-regulated and down-regulated DEGs

Up-regulated DEGs were significantly enriched in the Notch signaling pathway (aml04330; Fig. 5). The Notch signaling pathway controls the fate of adult hair follicular stem cells in the bulge. We found that down-regulated DEGs were enriched in four pathways (Fig. 5). These were the ribosome pathway (aml03010), thermogenesis pathway (aml04714) oxidative phosphorylation (aml00190) and ferroptosis (aml04216). The thermogenesis pathway is the child term of environmental adaptation. Oxidative phosphorylation (aml00190) is the downstream term of thermogenesis. Ferroptosis (aml04216) is a regulated form of cell death.

### Expression of hair growth-associated genes

We identified 28 hair growth-associated genes with altered expression (Table 1). *DUSP3, GNG5, RAC1*, and *TGFB1* were found to be highly down-regulated while the other genes were all up-regulated. These genes belonged to five signaling pathways, being Notch, Wnt, TGF-β, Mapk, and PI3K-Akt signaling pathways. *NOTCH1* (notch receptor 1) and *DTX4* (deltex E3 ubiquitin ligase 4) participate in the Notch signaling pathway. *DVL1* (dishevelled segment polarity protein 1), *DVL2* (dishevelled segment polarity protein 2), *FZD8* (frizzled class receptor 8), *NFATC2* (nuclear factor of activated T cells 2), and *VANGL2* (VANGL planar cell polarity protein 2) are linked to the Wnt signaling pathway. *CDKN2B* (cyclin dependent kinase inhibitor 2B), *SMAD3* (SMAD family member 3), *SMAD7* (SMAD family member 7), and *TGFB1* (transforming growth factor beta 1) are members of the TGF-β signaling pathway. *DUSP4* (dual specificity phosphatase 4), *DUSP7* (dual specificity phosphatase 7), *IKBKG* (inhibitor of nuclear factor kappa B kinase regulatory subunit gamma), and *RAC1* (Rac family small GTPase 1) function within the Mapk signaling pathway. Lastly, *IL6R* (interleukin 6 receptor), *OSM* (oncostatin M), and *RXRA* (retinoid X receptor alpha) are associated with the PI3K-Akt signaling pathway.

**Table 1:** Hair growth-associated gene list

Gene Symbol	logFC	Pathway
*CACNA2D2*	1.711396422	MAPK signaling pathway
*DUSP3*	-1.790143776	MAPK signaling pathway
*DUSP4*	1.566551716	MAPK signaling pathway
*DUSP7*	1.039509057	MAPK signaling pathway
*GADD45A*	1.305880229	MAPK signaling pathway
*MAPK8IP3*	1.740574574	MAPK signaling pathway
*IKBKG*	1.016803027	MAPK signaling pathway, PI3K-Akt signaling pathway
*DTX4*	1.406510433	Notch signaling pathway
*NCOR2*	1.058483914	Notch signaling pathway
*NOTCH1*	1.974692678	Notch signaling pathway
*DVL1*	1.189063569	Notch signaling pathway, Wnt signaling pathway
*DVL2*	1.066374756	Notch signaling pathway, Wnt signaling pathway
*CREBBP*	1.355427883	Notch signaling pathway, Wnt signaling pathway, TGF-beta signaling pathway
*GNG5*	-1.094827416	PI3K-Akt signaling pathway
*IL4R*	2.197209802	PI3K-Akt signaling pathway
*IL6R*	1.722598452	PI3K-Akt signaling pathway
*OSM*	1.108241336	PI3K-Akt signaling pathway
*RXRA*	1.251346341	PI3K-Akt signaling pathway
*AMHR2*	1.714991995	TGF-beta signaling pathway
*CDKN2B*	1.438709339	TGF-beta signaling pathway
*SMAD3*	1.973049459	TGF-beta signaling pathway
*SMAD7*	1.583980212	TGF-beta signaling pathway
*TGFB1*	-1.2528519	TGF-beta signaling pathway, MAPK signaling pathway
*CSNK2B*	-1.214386193	Wnt signaling pathway
*FZD8*	2.352985361	Wnt signaling pathway
*NFATC2*	1.369368061	Wnt signaling pathway
*VANGL2*	1.549672091	Wnt signaling pathway
*RAC1*	-1.175894859	Wnt signaling pathway, MAPK signaling pathway, PI3K-Akt signaling pathway

### Protein–protein interaction network of hair growth-associated DEGs

**Fig. 6:**
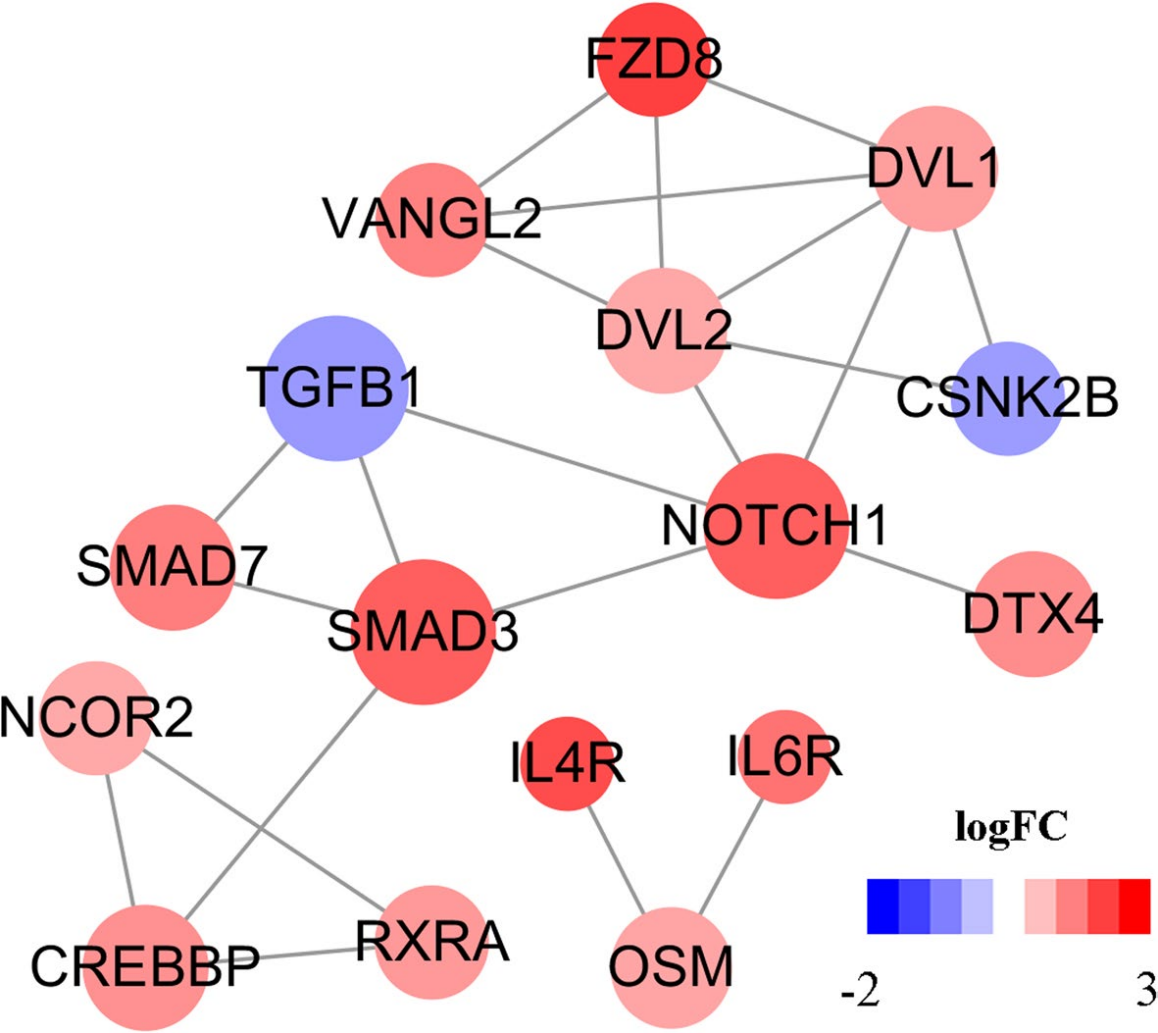
Protein–protein interaction network of DEGs. The red nodes represent up-regulated proteins. The blue nodes represent down-regulated proteins

We converted hair growth-associated DEGs into proteins using STRING. After removing 12 isolated nodes, a total of 22 interaction edges between 16 nodes were extracted from the database. Functional enrichment of STRING local network cluster was then constructed. The genes were significantly enriched in 13 clusters, including the Notch signaling pathway (CL:18,099), the TGF-β signaling pathway (CL:17,598), and the Wnt signaling pathway (CL:17,371). We calculated the hub genes using cytoHubba and obtained a score of 36 genes using EcCentricity algorithm (Additional file 5: Table S5). The top three genes were *NOTCH1, SMAD3, and TGFB1*. These three genes were the candidate genes associated with alopecia in male giant pandas. We plotted the hub genes interaction network (Fig. 6).

### Real-time quantitative PCR (qRT-PCR) validation

**Fig. 7:**
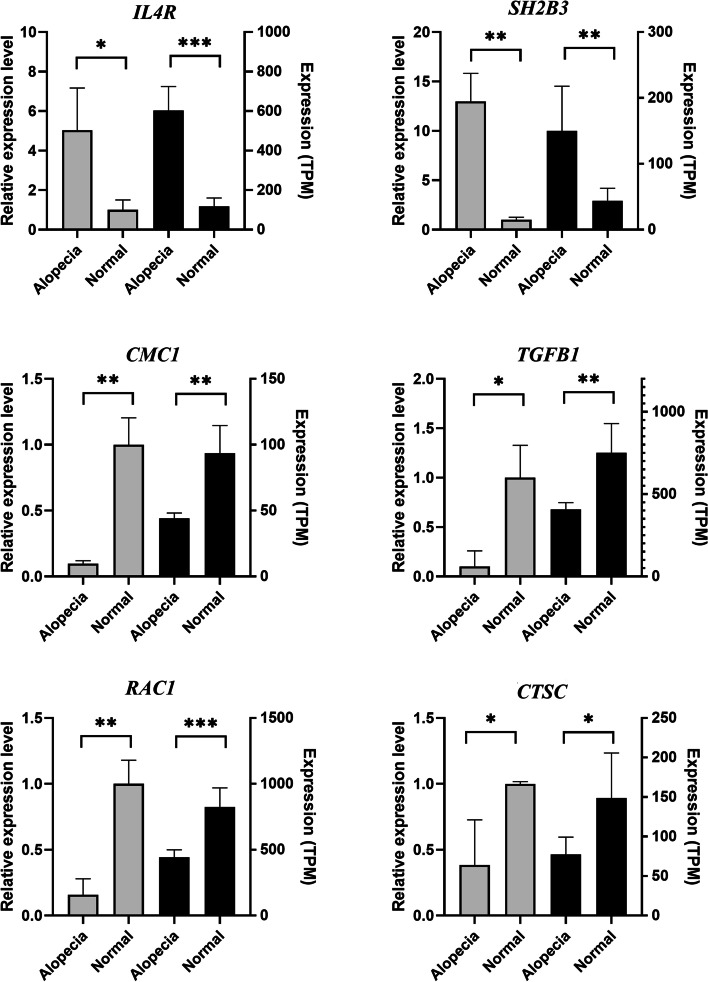
The expression level of genes verified by qRT-PCR. “*” represents the *P*-value < 0.05, “**” represents the *P*-value < 0.01, “***” represents the *P*-value < 0.001. Data were shown as mean ± SD. The left axis represents gene expression levels verified by qRT-PCR. The right axis represents the expression levels in TPM units of RNA-seq

We selected six DEGs (*IL4R, SH2B3, CMC1, TGFB1, RAC1, CTSC*) for verification. The results of qRT-PCR indicated similar expression tendencies with transcriptome sequencing (Fig. 7). The qRT-PCR validation improves the reliability of the present study.

## Discussion

Hair is a unique character in mammals, and has important biological functions, such as mate attraction, skin protection, and social communication [1, 31]. Alopecia is a common phenomenon in mammal, including humans, and can seriously affect health. Here we monitored the expression of hair growth-related genes based on peripheral blood transcriptomes of giant panda. We identified 28 hair growth-related genes with altered expression in alopecia male giant pandas compared to normal male pandas.

The GO term enrichment of “ribosome” and “structural constituent of ribosome”, and KEGG pathway enrichment of “ribosome” were observed in down-regulated genes. Several studies have identified a connection between the ribosome and hair growth [32–36]. Lv et al. found that the ribosome pathway was the most significantly enriched pathway by skin transcriptome analysis and may be related to the development and the density of secondary wool follicles in Chinese Merino sheep [32]. By miRNA sequencing and differentially expressed miRNAs analysis, the ribosome pathway may participate in dermal papilla cells viability and proliferation, and thereby affects hair cycling [34]. Plowman et al. showed that ribosomal proteins were detected in dissected portions from 30 hair follicles by Gel-free proteomic analysis [35]. In patients with severe active alopecia areata, the ribosome pathway was significantly enriched by functional annotation analysis [36]. The ribosome may influence protein synthesis that is important for human dermal papilla cells [33]. The oxidative phosphorylation pathway was down-regulated in giant pandas with alopecia. Oxidative phosphorylation can produce ATP for hair follicle stem cells differentiation and proliferation [37]. Our enrichment results are consistent with a previous study where the oxidative phosphorylation pathway was reported to be related to hair follicle cycling [34]. The thermogenesis pathway was another enriched pathway in our study, which was unusual of the hair follicle cycle activities, underlining the relationship between environmental temperature and hair growth [38]. The present study confirmed the abnormal expression of several pathways related to hair growth.

### Notch signaling pathway

The Notch pathway was found to be related to Hu sheep hair follicle growth and development when the source genes of differentially expressed circRNAs were compared between small waves and straight wool [39]. Additionally, the results of microRNA expression profiles showed that the Notch pathway may be an important regulator in the process of coat color formation in the goat (*Capra hircus*) [40]. In the current study, the Notch signaling pathway was significantly enriched in alopecia giant pandas. Notch is a local cell-signaling pathway and comprises evolutionarily conserved transmembrane receptors and has three possible functional roles in hair follicle development: lateral inhibition, boundary formation and lineage decision [41]. In the bulge, Notch signaling is required for follicular fate selection of adult hair follicle stem cells during hair follicle morphogenesis [41]. In the hair bulb, Notch controls the proper development of the hair shaft and inner root sheath [42]. Notch signaling cannot only suppress TGF-β to provide an optimal matrix proliferating environment, but it can also activate Wnt family members to regulate hair follicle keratinocyte differentiation [41]. *NOTCH1* is expressed in the follicle bulb and outer root sheath [3]. Inactivation of Notch1 in adult mice is characterized by a premature entry into catagen and leads to alopecia [43]. The overexpression of NOTCH1 delays inner root sheath differentiation and results in hair shaft abnormalities [44]. *NOTCH1* was highly expressed and up-regulated (about fourfold) in giant pandas with alopecia. *DTX4* (deltex E3 ubiquitin ligase 4) encodes Deltex protein that is a positive regulator of Notch signaling [45]. *DTX4* was also up-regulated in alopecia giant pandas. Therefore, alopecia in our giant pandas may be related to dysregulation of Notch signaling and *NOTCH1* may be the candidate gene.

### Wnt signaling pathway

The Wnt signaling pathway includes canonical Wnt/β-catenin, Wnt/PCP, and Wnt/Ca^2+^ signaling pathways [46]. In the canonical Wnt pathway, the Dvl protein is a key component of Wnt signaling and is recruited by Frizzled receptors and LRP co-receptors, preventing the constitutive destruction of cytosolic β-catenin [41, 46, 47]. The stabilization of β-catenin may play a role in hair follicle development [41]. Wnt signaling was the earliest signal in the development of the epidermis and has been found in the embryonic skin of mice [4]. The overexpression of DKK1, a Wnt inhibitor, could prevent the formation of hair follicles in mice [48]. The miR-29 family was reported to inhibit the Wnt and BMP signaling pathways causing eventual hair loss and alopecia [49]. The Wnt was reported to be involved in the hair follicle cycle in yaks (*Bos grunniens*) via mRNA and lncRNA analysis [50, 51]. *FZD8* (frizzled class receptor 8) as Wnt family receptor, was found to be expressed in dermal papillae cells that controlled feather regeneration in chickens [52]. *FZD8* was up-regulated in the induction of dermal fibroblasts into dermal papilla cell-like cells in nude mice, suggesting the important role of *FZD8* and the Wnt signaling pathway in hair follicle regeneration [53]. *DVL1* and *DVL2* as the key regulators of Wnt signaling, were up-regulated 2.3-fold and 2.1-fold, respectively. Overexpression of *DVL2* in the outer root may affect hair growth and structure, resulting in short hair [54]. Lee et al. found CXXC-type zinc finger protein 5 interacted with Dvl protein via its C-terminal Dvl-binding motif to negatively regulate hair regrowth [55]. In the Wnt/Ca^2+^ signaling pathway, *NFATC2* (nuclear factor of activated T cells 2) was up-regulated in giant pandas with alopecia. The inhibition of the nuclear factor of activated T cells pathway could enhance hair growth in follicular keratinocytes [56].In the Wnt/PCP signaling pathway, *VANGL2* (VANGL planar cell polarity protein 2) could drive the anterior-directed tilt of hair follicle placodes during early stages of hair follicle morphogenesis [57]. In vitro evidence showed that *VANGL2* plays a role in hair follicle polarization and orientation [58]. Our findings implied that the Wnt signaling pathway was the candidate pathway in giant panda alopecia syndrome.

### TGF-β signaling pathway

TGF-β signaling could activate intracellular effectors such as Smad proteins and kinases via ligands (TGF-β1/2/3, activins, BMPs, GDFs) bind to receptors (BMPR, TGFBR), controlling the activities of key transcription factors that promote epithelial differentiation [59]. TGF‐β was reported to regulate various biological processes in the skin, including the inhibition of keratinocyte proliferation and the stimulation of epithelial cell apoptosis [5]. During the hair follicle cycle of mice and humans, TGF‐β contributes to the induction of catagen [5]. Western blot analysis showed that the delayed hair regression was associated with a significant decrease in the expression levels of TGFB1, and high expression of TGFB1 in the epidermis resulting in the inhibition of normal skin development [60]. Liu et al. found that chronic expression of TGFB1 led to alopecia in adult transgenic mice [61]. The down-regulation of *TGFB1* in the giant panda may imply their partial responsibility for giant panda alopecia. All-*trans*-retinoic acid could activate the phosphorylation of Smad2/3, inducing apoptosis of dermal papilla cells [62]. Low levels of phosphorylated Smad2/3 delay the hair cycle in Tsukushi-null mutant mice [63]. *SMAD7* as an inhibitor of TGFβ signaling, was up-regulated in mice with alopecia [61], which was consistent with our findings in giant pandas with alopecia. Overexpression of Smad7 was responsible for aberrant hair follicle morphogenesis and hyperproliferation in the epidermis, leading to severe pathological alterations in transgenic mice epithelial tissues [64]. In addition, *CDKN2B* (cyclin dependent kinase inhibitor 2B) is a TGFβ target gene and encodes the cell-cycle inhibitor protein, and may be involved in hair follicle cycle [65]. Our data showed that several hair growth-related genes in TGFβ signaling were abnormally expressed in male giant pandas with alopecia.

### Mapk signaling pathway

MAPK (mitogen-activated protein kinase) comprises at least three groups, ERK (extracellular signal-related kinases), JNK (Jun amino-terminal kinases), and p38, and MAPK is involved in the regulation of normal cell proliferation, survival, differentiation, and migration [66]. Corticotropin‐releasing hormone has been reported to induce alopecia via the MAPK signaling pathway [67]. MAPK was suggested to regulate the quiescence of hair follicle stem cells and control the hair cycle [6]. *IKBKG* (inhibitor of nuclear factor kappa B kinase regulatory subunit gamma, also known as NEMO, IKKγ) is a downstream messenger of MAPK and necessary for full activation of NF-κB [68]. Mice lacking IKKγ or IKKβ develop severe inflammatory skin diseases, suggesting a significant role of NF-κB and IKKγ [69]. The *IKBKG* gene is found mutated in hypohidrotic ectodermal dysplasia, accompanied by variable degrees of alopecia [70]. ERK signaling is involved in the proliferation of the hair matrix cell and dermal papilla cell [71, 72]. *DUSP4* (dual specificity phosphatase 4) and *DUSP7* (dual specificity phosphatase 7) were upregulated in giant pandas with alopecia. *DUSP4* and *DUSP7* are two members of mitogen-activated protein kinase phosphatases family and are involved in dephosphorylation of ERK and negatively regulate MAPK [73, 74]. JNK functions in the proliferation and differentiation of bulge hair follicle stem cells. Hyperactivation of the JNK was associated with alopecia and topical application of the JNK inhibitor reverted faster hair growth [75, 76]. *RAC1* (Rac family small GTPase 1) is associated with JNK to initiate various cellular responses and maintains the differentiated state of hair follicle keratinocytes [77]. The deletion of *RAC1* resulted in the rapid depletion of stem cells in the adult mouse epidermis [78]. *RAC1* functions in keratinocyte proliferation and migration, and the inhibition of *RAC1* impairs the proliferation of keratinocytes [79]. The down-regulation of RAC1 may be the cause of alopecia in giant pandas.

### PI3K-Akt signaling pathway

Inhibition of PI3K or Akt noticeably suppressed hair follicle regeneration, suggesting a role of the PI3K-Akt signaling pathway in hair follicle de novo regeneration [7]. The activation of PI3K signaling can promote the proliferation and migration of dermal papilla cells [80]. Cai et al. found that lncRNA5322 could promote the proliferation and differentiation of hair follicle stem cells via the PI3K‑AKT signaling pathway [81]. Liu et al. found that in Rex rabbits ocu-miR-205 altered PI3K-Akt, Wnt, and Notch signaling and promoted hair follicle transition from the growth phase to the regression and resting phase [82]. *OSM* (oncostatin M) and *IL6R* (interleukin 6 receptor) were significantly up-regulated in giant pandas with alopecia. *OSM*, belonging to IL-6 family, negatively regulates hair growth and increased expression of *OSM* might contribute to alopecia [83, 84]. Kwack et al. found that IL-6 was upregulated in dermal papilla cells from balding patients [85]. *RXRA* (retinoid X receptor alpha) is highly expressed in skin and in the hair follicle outer root sheath [86]. The mutant mice without *RXRA* developed hair follicle degeneration, then alopecia [87]. Blocking retinoic acid signaling delayed anagen initiation of hair follicle, while increasing retinol accelerated the transition from telogen to anagen [88]. Therefore, we conclude that the PI3K-Akt signaling pathway might also be a candidate pathway in giant pandas with alopecia.

## Conclusion

We investigated the blood transcriptome expression profiles of four alopecia pandas and seven healthy giant pandas. A total of 458 up-regulated DEGs and 211 down-regulated DEGs were identified. We obtained 28 hair growth-related genes with altered expression, and identified three hub genes *NOTCH1*, *SMAD3*, and *TGFB1* in our PPI analysis. Five hair growth-related signaling pathways with abnormal expression were identified, being Notch, Wnt, TGF-β, Mapk, and PI3K-Akt. In conclusion, giant panda alopecia may be complex, involving abnormal expression of multiple genes and pathways. Our study provides a foundation for further studies in prevention and treatment strategies for giant pandas with alopecia.

## Supplementary Information

**Additional file 1: Table S1.**

**Additional file 2: Table S2.**

**Additional file 3: Table S3.**

**Additional file 4: Table S4.**

**Additional file 5: Table S5.**

## Data Availability

Raw sequence data are accessible at NCBI under the BioProject accession number PRJNA747307 (https://www.ncbi.nlm.nih.gov/sra/PRJNA747307).

## Abbreviations

HF: Hair follicle
DEGs: Differentially expressed genes
TPM: Transcripts per million
PCA: Principal components analysis
FC: Fold change
FDR: False discovery rate
GO: Gene Ontology
KEGG: Kyoto Encyclopedia of Genes and Genomes
